# Controllable release of pirfenidone by polyvinyl alcohol film embedded soft contact lenses *in vitro* and *in vivo*

**DOI:** 10.1080/10717544.2021.1895911

**Published:** 2021-03-27

**Authors:** Caiqing Wu, Ping Wai Or, Jones Iok Tong Chong, Isuru K. K. Pathirage Don, Ching Hymn Christopher Lee, Kaili Wu, Minbin Yu, David C. C. Lam, Yangfan Yang

**Affiliations:** a State Key Laboratory of Ophthalmology, Zhongshan Ophthalmic Center, Sun Yat-sen University, Guangzhou, China; b Department of Mechanical and Aerospace Engineering, The Hong Kong University of Science and Technology, Kowloon, Hong Kong

**Keywords:** Glaucoma, soft contact lens, pirfenidone, sustained delivery of drug

## Abstract

To increase the amount of pirfenidone (PFD) loaded in polyvinyl alcohol (PVA) film embedded soft contact lens (SCL), and evaluate its function of sustaining delivery of drug *in vitro* and *in vivo*. Drug loading efficiency within PVA film and SCLs, drug release from SCLs *in vitro*, and the effects of parameters of SCLs and external environment on drug release *in vitro* were evaluated by ultraviolet–visible spectrophotometer at 312 nm. Safety of SCLs was evaluated *in vitro* by transformed human corneal epithelial cell. Safety *in vivo* was determined by optical coherence tomography and histology of anterior segment of rabbits. Drug release study in tear fluid and aqueous humor were measured by ultra-performance liquid chromatography. SCLs had smooth surface and were fit for experimental rabbits. Amount of PFD in PVA film and SCLs were 153.515 μg ± 12.508 and 127.438 μg ± 19.674, respectively, PFD in PVA film was significantly higher than SCLs (*p*=.006) and closed to 150 μg (targeting amount of PFD to be loaded). Thickness of SCLs, molecular weight of PVA, and amount of PVA used in SCLs affected drug release *in vitro* significantly. Thickness of PVA film and amount of drug in SCLs had no effect on drug release rate *in vitro*. SCLs were safe *in vitro* and *in vivo*, PFD released from SCLs could be detected around 12 hours in tears and aqueous humor, and the concentration of drug was higher than eye drop at all detected time points while amount of PFD in SCLs was lower than eye drop. Drug loaded PVA film embedded SCLs may be a promising ocular drug delivery system.

## 1. Introduction

Glaucoma, an ocular disease that progressively damages optical nerve, is one of the leading causes of blindness worldwide. The number of people with glaucoma was estimated to increase to 111.8 million in 2040 (Tham et al., 2014). Surgery to reduce the intraocular pressure is required when the intraocular pressure was not satisfactory under the treatment of medicine. However, wound healing after glaucoma filtration surgery is the most common cause of surgical failure (Zada et al., 2018). Clinically available anti-fibrosis drugs, such as 5-fluorouracil (5-FU) and mitomycin C (MMC) are limited for their severe complications (Aref, 2016). There is an urgent need for an alternative medicine to reduce the postoperative scar formation.

Pirfenidone (PFD) was shown to have anti-fibrosis effect on many tissues and organs in the human body (Yamazaki et al., 2012; Flores-Contreras et al., 2014; King et al., 2014). Previous study showed PFD eye drop (0.5% w/v) possessed good ocular tissue permeability and had safe and effective anti-fibrosis efficiency after ocular filtration surgery (Zhong et al., 2011), however, because of the ocular anatomy, PFD fluid had poor ocular bioavailability like other ophthalmic solutions (Sun et al., 2011).

A study showed that the safe and effective dosage of PFD eye drop for the treatment of wound healing after glaucoma filtration surgery was six times a day (six drops = 300 μl = 1500 μg per day). Since bioavailability of eye drop is <5%, the absorbed amount is estimated to be 75 μg per day for the postoperative fibrosis treatment of eyes (Zhong et al., 2011). Ocular bioavailability of drug loaded in soft contact lens (SCL) was >50% (Ross et al., 2019; Maulvi et al., 2020; Xue et al., 2020), so the required dose for SCLs will be approximately 150 μg per day, which was our targeted amount of PFD in SCLs.

In our previous research, we used polyvinyl alcohol (PVA) which possessed relative higher oxygen permeability and lower protein adsorption and silicone that had high oxygen permeability to make SCLs, and showed the fabricated SCLs could prolong retention time of PFD *in vitro* and *in vivo* with a relative lower drug capacity (Hyon et al., 1994; Tran & Yang, 2019; Wu et al., 2020).

In this study, the drug capacity of our fabricated PFD loaded PVA film embedded SCLs was improved and its drug delivery efficiency was explored *in vitro* and *in vivo*.

## 2. Materials and methods

### 2.1. Materials

Pirfenidone (≥98% GC) was purchased from Acmec Biochemical (Tokyo Chemical Industry, Tokyo, Japan). Medical grade PVA (Mw = 25 kDa, 78 kDa, 146–148 kDa) obtained from Life Sciences (New York, NY). Liquid silicone elastomer MED-6015 of medical grade was purchased from NuSil Technology LLC (Carpinteria, CA). Polyvinylpyrrolidone 360 (PVP360, average mol wt 360,000) was purchased from Sigma-Aldrich (St. Louis, MO). Transformed human corneal epithelial cell (HCE-T) lines were from RIKEN Cell Bank (Tsukuba, Japan). DMEM/F12, fetal calf serum, penicillin–streptomycin for cell culture, and phosphate buffer saline were from Gibco (Thermo Fisher Scientific, Suzhou, China). Insulin and epithelial growth factor were gained from Sigma-Aldrich Chemicals (St. Louis, MO). Acetonitrile of HPLC grade was purchased from Aladdin (Beijing, China).

### 2.2. Fabrication of PFD-PVA film embedded silicone contact lenses

The procedures of fabricating PFD loaded PVA film were described as previously with a small change (Wu et al., 2020), Briefly, 105 mg PFD and 2 g PVA were dissolved and stirred homogenously in deionized water and then was poured on a fiber-free and oil-free acrylic substrate to cure a 100 μm sheet in 60 °C oven, that is, the PFD loaded drug insert. A crescent insert was punched mechanically and then precisely weighed for targeted drug loading. It was then embedded into two layers of around 45 μm silicone films to form SCLs. The SCLs was treated with oxygen plasma (Branson IPC 3000 O2 Asher) for one minute each side to make the lens surface hydrophilic and soaked in PVP360 solution for one minute (Lai et al., 1996). The drug-loaded contact lens was ultra-violet (UV) radiated using a UV-C lamp (TUV T8, Philips, Amsterdam, Netherlands) at a peak 253.7 nm for 30 minutes (Harris et al., 1993).

### 2.3. PFD loading in drug insert

In order to verify loaded amount of PFD in SCLs, the drug loading capacity within the PVA film was first determined. Precisely weighted PFD loaded PVA films were immersed in PBS buffer, the films could be dissolved within several seconds, and the quantity of PFD loaded in PVA films were evaluated by a UV–visible spectrophotometer (BioSpectrometerbasic, Eppendorf, Germany) at 312 nm according to multi-wavelength scan and calibration curve after being diluted.

### 2.4. PFD loading capacity within SCLs and release from SCLs *in vitro*

PFD released from contact lenses *in vitro* were carried out as reported previously with a small change (Yang et al., 2016). SCLs were soaked in 24-well plates with 2 ml release medium in thermostatic oscillator (MaxQ 400, Thermo Scientific, Waltham, MA) shaking at 60 rpm at certain temperatures, 2 ml release medium was extracted at predetermined time intervals (0.25 h, 0.5 h, 1 h, 4 h, and 8 h) for evaluating released amount of PFD with the UV spectrophotometer at 312 nm, after dabbed with a Kimwipe, fresh 2 ml PBS was replaced during the specific time intervals. The measurement was repeated until no PFD was detected. The total amount of released PFD in SCLs was regarded as the loading capacities of SCLs (Hiratani et al., 2005). Effects of parameters of SCLs on drug release *in vitro* were explored when release medium was PBS and temperature was 37 °C. The effect of environment temperatures (25 °C, 37 °C, and 60 °C) and release mediums (PBS buffer and simulated tear fluid that was made of 6.78 g/l NaCl, 2.18 g/l NaHCO_3_, 0.084 g/l CaCl_2_·2H_2_O, and pH 7.4) on drug release *in vitro* were also explored (Maulvi et al., 2016).

### 2.5. Cytotoxicity test

HCE-T cells were used for toxic test *in vitro* whose complete medium consisting of DMEM/F12, 10% fetal calf serum, 1% penicillin–streptomycin, 5 μg/ml insulin, and 10 ng/ml epithelial growth factor (Dutot et al., 2013). SCLs samples were shred into small pieces and immersed in 2 ml of DMEM/F12 for 24 hours in cell incubator at 37 °C supplied with 5% CO_2_ to produce SCLs medium as experimental group. Around every 5000 cells were transferred to 96-well plates, then the medium of cells was replaced with filtered experimental DMEM/F12 after starving cells for 12 hours by DMEM/F12. Incubating cells for consecutive 12, 24, 48, 60, and 72 hours, the culture medium was removed and washed cells three times with PBS. One hundred microliters medium containing 10 μl Cell Counting Kit-8 (CCK-8, Dojindo, Kumamoto, Japan) reagent was used to incubate cells for 2 h at 37 °C and the absorbance of the medium was measured at 450 nm with microplate reader (BioTek, Winooski, VT). Cell viability was estimated by CCK-8 Kit.

### 2.6. Animal studies

Female New Zealand white rabbits weighing between 2.5 kg and 3.0 kg were kept individually in standard cages in a light-controlled room at 20 ± 1 °C and 50 ± 5% relative humidity, with no restriction of food or water intake. All animal experiments conformed with the principal of ARVO (Association for Research in Vision and Ophthalmology) statements. All animal procedures were approved by the ethics committee of animals in Zhongshan Ophthalmic Center (SYXK2020-0058).

#### 2.6.1. Safety study in vivo

Right eyes of six rabbits were treated with SCLs and the thickness of cornea were evaluated before and after wearing SCLs for seven consecutive days by optical coherence tomography (Spectralis OCT, Heidelberg, Germany). After the SCLs treatment, the rabbits were euthanized by overdosed anesthetics. Eyes were immersed in 4% paraformaldehyde after removed from bodies instantly, then dehydrated with gradient alcohol, cleared by xylene and embedded with paraffin. Experimental eyes were gained and processed for hematoxylin–eosin (H&E) staining according to manufactural procedures (Dixon et al., 2018).

#### 2.6.2. Release study of drug loaded soft contact lenses in tears

Thirty microliters of 0.5% PFD solution was dripped into conjunctival sacs of rabbits with pipette and served as control. No anesthetization was applied during application of SCLs. After PFD treatment, 3 μl tear fluid was extracted from lower marginal by 5 μl quantitative disposable capillaries at predetermined time intervals (5 min, 10 min, 20 min, 0.5 h, 1 h, 2 h, 4 h, 6 h, 8 h, and 12 h). The extracted tear fluid was flushed three times with 37 μl deionized water in 200 μl tubes. Ten microliters 10% perchloric acid was added and mixed thoroughly to precipitate protein. The mixture was centrifuged at 12,000 rpm for 15 minutes and the supernatant was taken out for PFD detection by ultra-performance liquid chromatography (UPLC) according to standard calibration curve.

#### 2.6.3. Release study of drug loaded contact lenses in aqueous humor

At every predetermined time points after treated with eye drop (1 h, 2 h, and 4 h) or SCLs (1 h, 2 h, 4 h, 6 h, 8 h, and 12 h), six rabbits were killed at each time point by intravenous injection of pentobarbital (100 mg/kg). Twenty microliters aqueous humor was obtained through a 1 ml syringe at the limbus after the conjunctival sac was flushed with deionized water. Twenty microliters 10% perchloric acid was used to precipitate protein. Samples were centrifuged at 12,000 rpm for 15 minutes to remove protein and the supernatant was used to measure the concentration of PFD.

#### 2.6.4. Measurement of PFD concentration by UPLC

All the samples were stored at 4 °C before analysis of UPLC to evaluate concentration of PFD as a function of time and all the samples were measured under the same condition of UPLC. One microliter of each sample was used for detection of PFD by ACQUITY UPLC^®^ BEH C18 column (1.7 μm, 2.1 × 50 mm, Waters, Milford, MA). The UV–visible detector (ACQUITY UPLC H-Class, Philadelphia, PA) was set at 314 nm according to multi-wavelength scan. Forty-five percent acetonitrile (A) and 55% ultrapure water (C) were used as the mobile phase and the flow rate was 0.3 ml/min.

### 2.7. Statistical analysis

Results of this study were expressed in mean ± standard deviation (SD). Differences between two and more groups were evaluated with one-way ANOVA and two-way ANOVA test, respectively, by SPSS 25.0 software (SPSS, Inc., Chicago, IL). *p*<.05 was regarded as statistically significant difference between groups.

## 3. Results

### 3.1. PFD loading efficiency within the PVA film and SCLs

**Figure 1.**
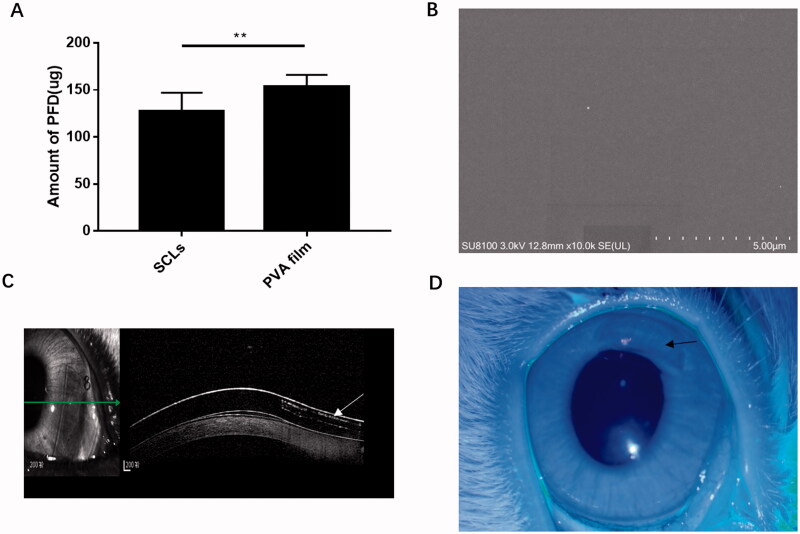
Amount of loaded drug and pictures of soft contact lenses in scanning electron microscope, coherence tomography scan, and eyes of rabbits. (A) *In vitro* measured amount of PFD loaded in PVA film and SCLs were 153.515 μg ± 12.508 and 127.438 μg ± 19.674, respectively, the data were plotted as mean ± SDs (*n* = 5 and 44, respectively) (**p<.01); (B) picture of scanning electron microscope of SCLs; (C) picture of coherence tomography scan of SCLs, the white arrow indicated the shape of PFD loaded PVA film; (D) picture of SCLs in eye of rabbit, the black arrow means the PFD loaded crescent area in SCLs.

As shown in Figure 1(A), amount of PFD in PVA film (*n* = 5) and SCLs (*n* = 44) were 153.515 μg ± 12.508 and 127.438 μg ± 19.674, respectively. The target amount of PFD in PVA film could be evaluated relatively precise through weighing; however, drug loading in SCLs was lower than drug insert. The percentage of PFD loss in SCLs was about 17.385%. The main reason for the drug loss was the aqueous environment during surface treatment. Figure 1(B) shows a scanning electron microscope of SCLs, which indicated the smooth surface of SCLs. Figure 1(C) shows the shape of PVA in SCLs under optical coherence tomography scan (white arrow). The picture of SCLs worn in eye of rabbit is shown in Figure 1(D) (black arrow indicated the transparent drug-loaded crescent).

### 3.2. Effects of parameters of soft contact lens on drug release *in vitro*

**Figure 2.**
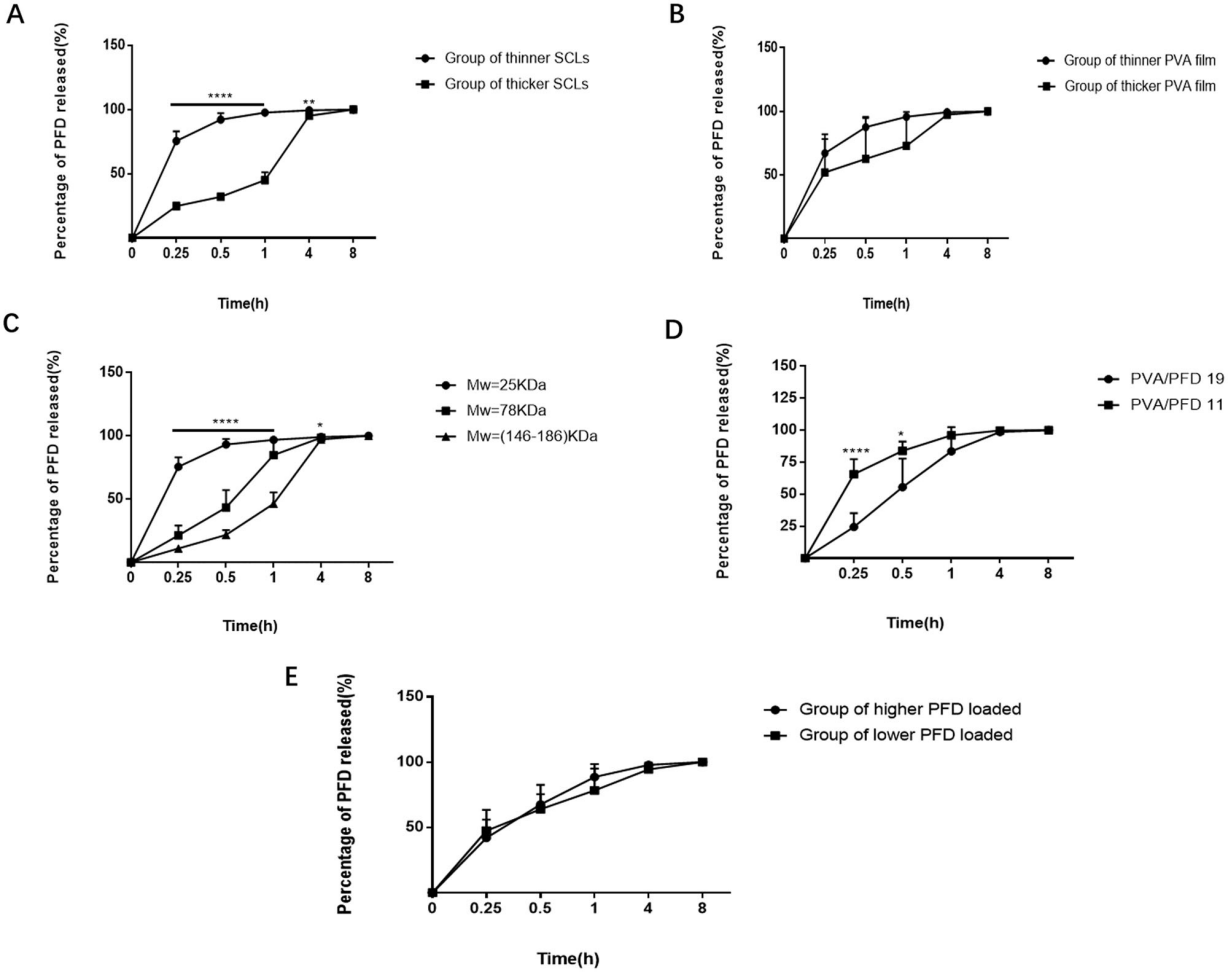
Effects of parameters of SCLs on drug release *in vitro*. (A) Drug release *in vitro* of different thickness of SCLs group; (B) drug release *in vitro* of SCLs with different thickness of PVA film; (C) drug release *in vitro* with different molecular weight of PVA; (D) the effect of amount of PVA on drug release *in vitro*; (E) the effect of different total amount of PFD in SCLs on drug PFD release *in vitro*. All above data were shown as mean ± SDs (*n* = 6) (*p<.05, **p<.01, ****p<.0001).

Parameters of SCLs involved in this work are shown in Table 1. Effects of thickness of SCLs (groups 1 and 2 in Table 1), thickness of PFD loaded PVA film (groups 3 and 4 in Table 1), molecular weight of PVA (groups 5, 6, and 7 in Table 1), ratio of PVA to PFD of SCLs (groups 8 and 9 in Table 1), and total amount of PFD loaded (groups 10 and 11 in Table 1) on drug release *in vitro* were studied. As shown in Figure 2(A), thickness of SCLs had statistically significant effect on drug release *in vitro* (*p*<.05). The thicker the SCLs were, the much slower the drug release was *in vitro* at all time points. Figure 2(B) indicates that thicker PFD loaded PVA film could slow down the release rate of PFD; however, thickness of PFD loaded PVA film had no obvious effect on drug release (*p*>.05). Figure 2(C) shows that molecular weight of PVA also had statically significant effect on rate of drug release *in vitro*. The smaller molecular of PVA was the faster rate of drug release had *in vitro* (*p*<.05). As shown in Figure 2(D), the ratio of PVA to PFD in drug insert affected drug release *in vitro* significantly, compared to ratio of 11, drug release rate of ratio of 19 was relatively slower, which maybe because PVA prevented PFD release from SCLs *in vitro*. Drug release rate of higher amount of PFD loaded group (105.575 μg ± 4.529) and lower amount of PFD loaded group (63.088 μg ± 27.091) had no difference at all time points (*p*>.05) (Figure 2(E)). In order to maximize PFD functional time on ocular surface to increase SCLs bioavailability. Following experiments were done with the SCLs made with larger molecular weight (146–186 kDa) of PVA and the ratio of PVA to PFD was 19.

**Table 1.** Parameters of soft contact lenses.

Group number	Thickness of contact lenses (μm; mean ± SD)	Thickness of PVA film (μm; mean ± SD)	Molecular weight of PVA (kDa)	Ratio of PVA to PFD	Amount of released PFD (μg; mean ± SD)
1	231.167 ± 5.492	99.500 ± 7.842	25	11	126.669 ± 14.606
2	167.333 ± 7.033	91.000 ± 17.006	25	11	129.421 ± 8.847
3	185.167 ± 18.841	75.667 ± 3.204	25	11	127.663 ± 5.265
4	210.333 ± 28.367	108.167 ± 5.636	25	11	124.978 ± 8.597
5	183.167 ± 19.934	84.000 ± 11.832	25	11	131.008 ± 7.665
6	184.000 ± 19.535	79.000 ± 4.561	78	11	153.183 ± 15.344
7	177.667 ± 20.520	87.000 ± 7.694	146–186	11	119.708 ± 9.752
8	183.000 ± 22.432	100.500 ± 11.095	25	19	123.621 ± 10.325
9	181.167 ± 18.433	92.500 ± 18.512	25	11	125.134 ± 9.160
10	173.833 ± 8.909	89.667 ± 19.511	78	19	105.575 ± 4.529
11	196.333 ± 25.828	119.833 ± 30.980	78	19	63.088 ± 27.091

SD: standard deviation.

### 3.3. Effects of external environment on drug release *in vitro*

**Figure 3.**
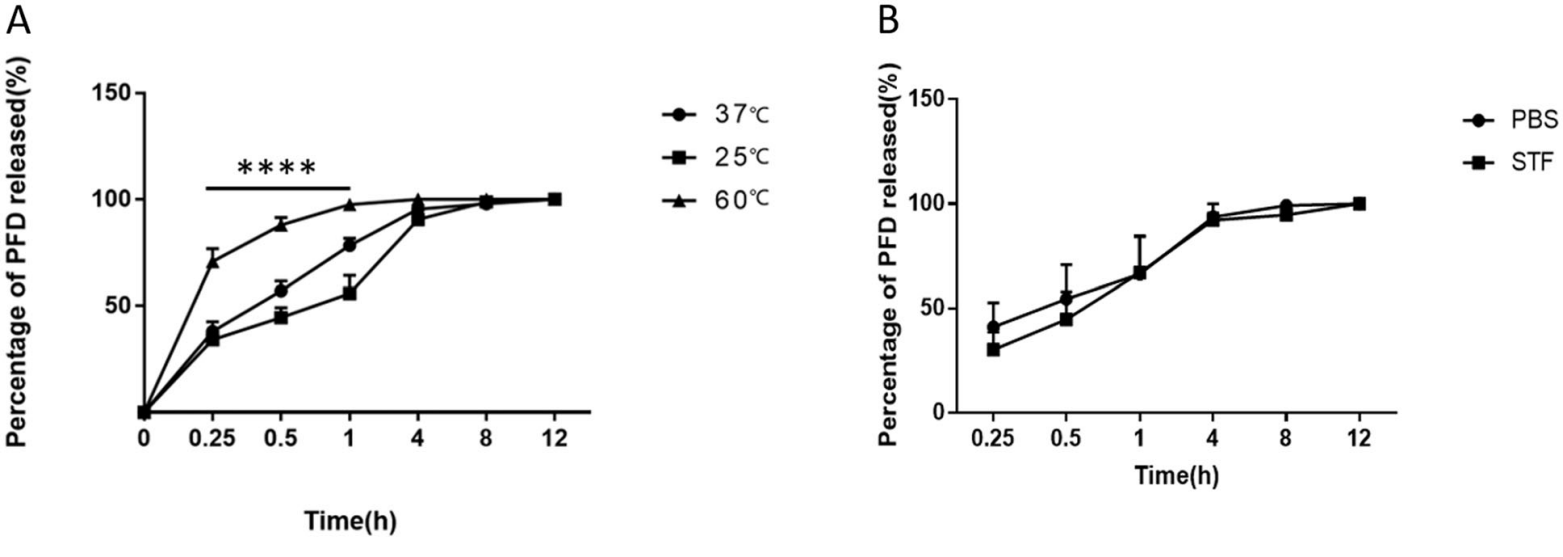
The effect of temperature and release medium on drug release *in vitro*. (A) Drug released from SCLs at different temperature, the data were described as mean ± SDs (*n* = 4) (****p<.0001); (B) drug release *in vitro* with release medium was PBS or STF, the data were depicted as mean ± SDs (*n* = 5).

SCLs were on eyes surfaces, ocular surface microenvironment can be affected by the external environment. The effect of temperature on PFD released from SCLs *in vitro* was explored. As can be seen from Figure 3(A), ambient temperature had a significantly effect on drug release *in vitro*. Release rate of PFD decreased with lower ambient temperature (*p*<.05). In order to better mimic microenvironment of human eyes, STF was used to substitute PBS to study drug release rate. However, changing of drug release medium had no relationship with drug release rate *in vitro* (Figure 3(B)).

### 3.4. Safety test *in vitro*

**Figure 4.**
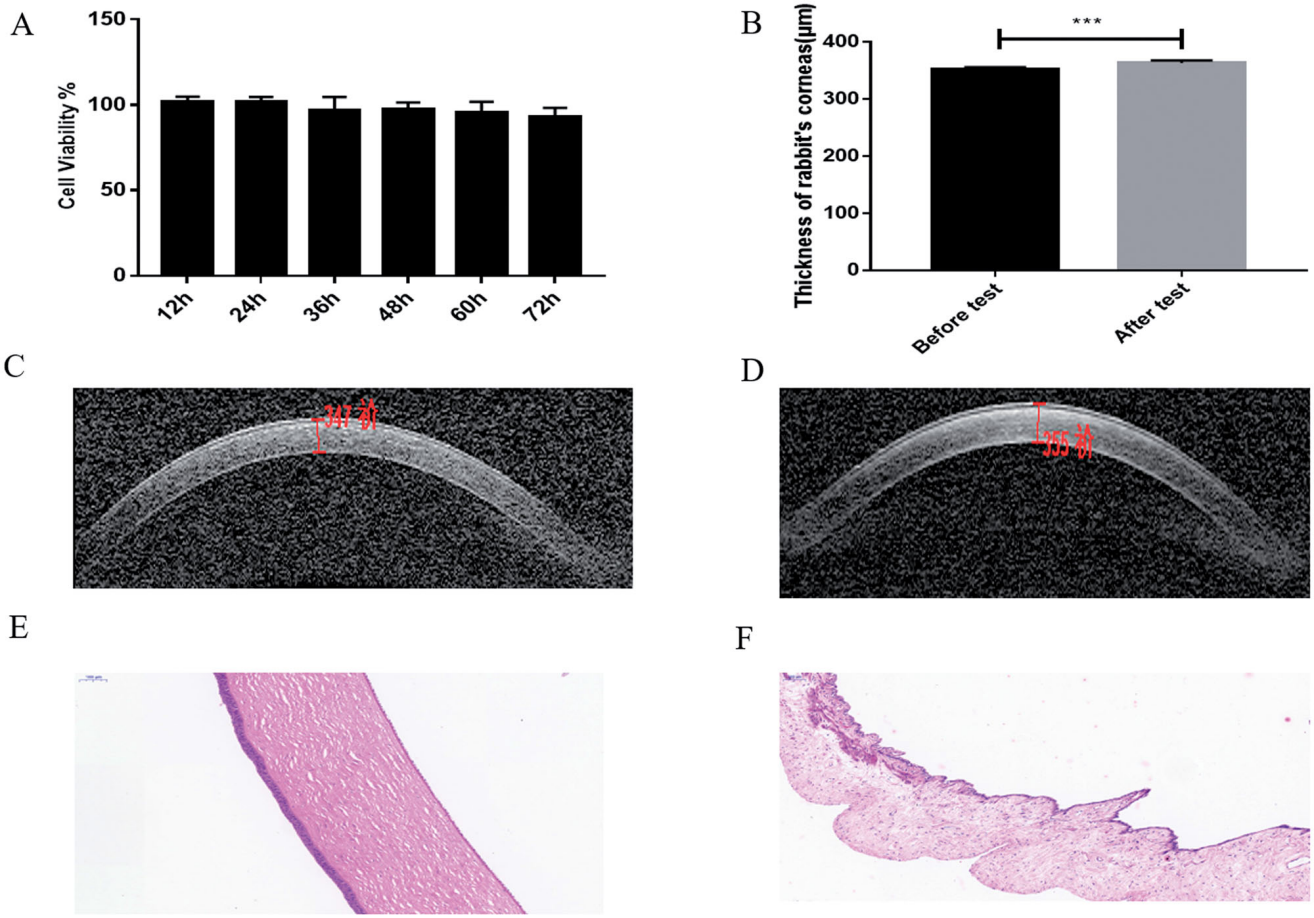
SCLs were safe *in vitro* and *in vivo*. (A) Cell viability of HCE-T cells were above 90% and had no significant change at all measured time points compared with control group; (B) the measured thickness of corneas of rabbits before and after safety test, the data was shown as mean ± SDs (*n* = 6,***P < .001); (C) picture of measured thickness of corneas of rabbits before the test (祄=μm); (D) picture of measured thickness of corneas of rabbits after the test (祄=μm); (E) histological picture of corneas of rabbits after wearing SCLs for consecutive seven days; (F) picture of histology of irises of rabbits after wearing SCLs for consecutive seven days. All the data were described as mean ± SDs (*n* = 6).

After treated with extraction of SCLs for (12, 24, 36, 48, 60, 72) hours, the cell viability of HCE-T cells was 101.992 ± 2.810, 101.387 ± 3.327, 96.890 ± 7.857, 97.454 ± 3.925, 95.209 ± 6.663, and 93.011 ± 5.342, respectively (Figure 4(A)). No significant difference was found after different times (*p*>.05). The above results showed the SCLs were safe *in vitro*.

### 3.5. Toxicity test in tissues

The fabricated SCLs could be wore more than seven days without any restriction of general activities of rabbits. As Figure 4(B) indicates, the thickness of cornea was much higher after wearing SCLs for consecutive seven days (day 0: 351.883 μm ± 4.708 vs. day 7: 363.500 μm ± 4.637, *n* = 6, *p*=.0006). Pictures of optical coherence tomography showed the thicker corneas after test (Figure 4(C,D)). However, histology test of corneas (Figure 4(E)) and irises (Figure 4(F)) have no significant toxin change, which mean that the SCLs were safe for rabbits after a long time for wearing (*n* = 6).

### 3.6. Drug release studies of contact lenses *in vivo*

**Figure 5.**
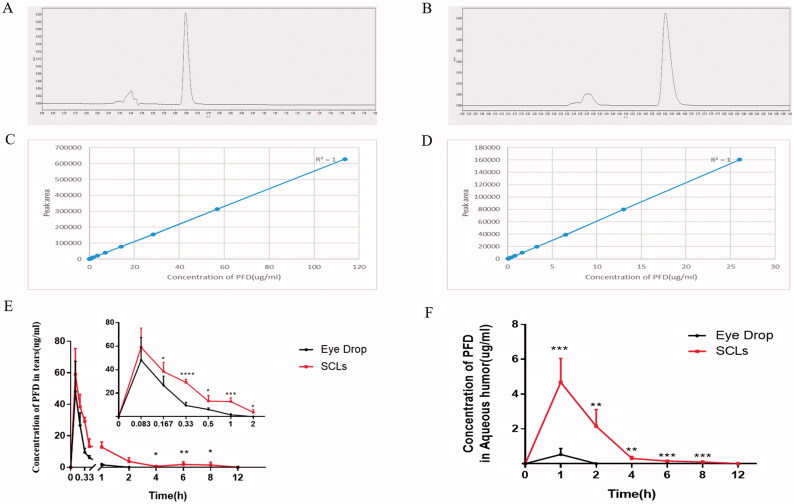
Fabricated SCLs could prolong duration of PFD in tears and aqueous humor with lower capacity of PFD. (A) Chromatogram of tears samples in UPLC; (B) chromatogram of aqueous humor samples in UPLC; (C) calibration curve of concentration of PFD in tears; (D) calibration curve of concentration of PFD in aqueous humor; (E) concentration of PFD in tears at different time points, the data were described as the mean ± SDs (*n* ≥ 6) (*p<.05,**p<.01, ***p<.001, ****p<.0001); (F) concentration of PFD in aqueous humor at different time points, the data were depicted as the mean ± SDs (*n* = 6) (**p<.01, ***p<.001).

The retention time of PFD was around 0.625 min in tears (Figure 5(A)) and aqueous humor (Figure 5(B)) samples. Calibration curve of PFD in tears and aqueous humor sample were linear over the concentration range from 0.028 μg/ml to 113.636 μg/ml (*R*^2^=1) (Figure 5(C)), and 0.051 μg/ml to 26.042 μg/ml (*R*^2^=1) (Figure 5(D)), respectively.

As we have mentioned above, the targeted capacity of drug was 150 μg, 30 μl of 0.5% eye drop in this work was used as control. After wearing the SCLs and administrating the eye drop in rabbits, PFD concentration in tears at different time intervals is shown in Figure 5(E). Results showed that the concentration of PFD released from the SCLs was higher than eye drop at all time points, and was significantly higher than that from eye drop after five minutes (*p*<.05). Compared to eye drop, even with a relatively lower amount of drug (153.515 μg ± 12.508 vs. 127.438 μg ± 19.674). The SCLs were shown to maintain at a higher concentration of drug for extended delivery, and PFD was found to stay in tears for nearly 12 hours. In Figure 5(F), PFD concentration in aqueous humor was also much higher than that of eye drop at all measured time points (*p*<.01). Compared with the eye fluid, which showed that PFD duration was around two hours, PFD released from the SCLs in aqueous humor could prolong to eight hours.

## 4. Discussion

Soft contact lenses, which could prolong retention time of drugs on eyes, increase the amounts of drugs released to cornea and improve drug bioavailability greatly. SCLs have been used as ocular drug delivery system for more than 50 years (González-Chomón et al., 2013). Also, there are various innovated SCLs for ocular drug delivery in recent years to improve bioavailability. Researchers have made important progress in prolonging functional time of drugs *in vitro* an *in vivo* (Maulvi et al., 2018; Gote et al., 2019; Bengani et al., 2020; Zhang et al., 2020). In this study, we increased drug loading capacity in PVA film embedded SCLs, and evaluated drug release *in vitro* and *in vivo*.

Consistent with other research results, compared with eye drop, SCLs significantly prolonged retention time of PFD *in vitro* and *in vivo* (Maulvi et al., 2018; Gote et al., 2019; Bengani et al., 2020; Zhang et al., 2020). Thicker SCLs slowed down drug release *in vitro*, because molecules of drug within SCLs need to travel much longer channels to arrive corneas when the SCLs were thicker. Molecular weight of PVA also had significant effect on drug release *in vitro*, it maybe because the longer molecular chain is more difficult to dissolve and increases viscosity of solvent (Halima, 2016). Larger ratio of PVA to PFD could also decrease release rate of PFD *in vitro*, which is because of resistance from molecules of PVA. Though there were many factors such as thickness of SCLs, molecular weight of PVA, etc., have impact on drug release rate *in vitro*, the total released time of PFD had no obvious alteration, which was around eight hours. Compared with eye drop, the curves of PFD released *in vivo* illustrated relatively smoother in SCLs, in spite of an initial burst release of PFD, which was in consistency with drug release *in vitro*.

Although the fabricated SCLs were thicker than commercial products, had a high initial burst release of PFD, and would be more costly and need to be changed daily compared to eye drops, there are still some advantages of them to be used as a suitable ocular drug delivery system. First, our present study showed increased drug loading capacity of PFD in SCLs compared with previous study (Wu et al., 2020), which means that the drug loading capacity could be changed with alteration of fabricating. Second, they were safe *in vitro* and *in vivo*. They were shown to wear for more than seven days without obvious toxicity, which means their suitable use for a long-time wear to delivery drugs. Moreover, drug release rate could be controlled by adjusting parameters of SCLs during make. Versatile fabrication of SCLs made it that could be loaded with more than one type of drugs and probably increases therapeutic efficiency.

## 5. Conclusions

Drug loading capacity could be improved in drug loaded PVA film embedded SCLs, and the SCLs could be a promising delivery system to controlled delivery ocular drugs.
